# Reading fiction in early adolescence predicts the ability to imagine our own future

**DOI:** 10.3758/s13423-026-02973-w

**Published:** 2026-07-29

**Authors:** Anna-Lisa Cohen, Nicholas Buttrick

**Affiliations:** 1 Department of Psychology, Yeshiva University, 2495 Amsterdam Ave, New York, NY 10033 USA; 2 University of WI–Madison, Madison, WI USA

**Keywords:** Episodic future thinking, Reading fiction, Future simulation, Cognitive flexibility

## Abstract

Episodic future thinking is the capacity to mentally simulate events that might occur in one’s future. Future thinking and reading fiction, most often studied separately, both rely on the construction and simulation of hypothetical imagined events. We guided participants to simulate an average day 10 years in the future. We predicted that the ability to generate alternatives (cognitive flexibility) and the tendency to become immersed by stories (transportability) would be positively associated with future simulations. Furthermore, we expected that higher habitual reading of fiction in adolescence would be positively associated with these traits. Results showed that greater reading of fiction in middle school, especially the type of fiction that allows readers to encounter different worlds, predicted higher degrees of both cognitive flexibility and transportability in the present day, and both traits were significantly associated with higher vividness and perceived control of the future.

**Supplementary Information:**

The online version contains supplementary material available at https://doi.org/10.3758/s13423-026-02973-w.

Anything you may hold firmly in your imagination can be yours

—William James (1892)

Episodic future thinking (EFT) refers to the ability to simulate experiences that might occur in one’s personal future (Atance & O’Neill, 2001). These simulations recombine fragments of past experiences and knowledge into novel and coherent scenarios, also known as mental time travel (Schacter & Addis, 2007; Suddendorf & Corballis, 1997). Imagining the future leads to numerous cognitive and emotional advantages such as better emotion regulation and more effective decision-making (see Schacter et al., 2017, for a review). Research on episodic future thinking has increased exponentially in recent years. Yet there is very little known about the mechanisms that underlie this human capacity. In the present study, we identify two traits that we propose are positively associated with episodic future thinking: *cognitive flexibility* (the ability to switch cognitive sets in response to changing environmental demands) and *transportability* (a dispositional tendency to become immersed by stories). We suggest that both factors contribute to an increase in the vividness of future simulations which leads to an increased sense of perceived control, the personal agency one experiences while imagining a future scenario (Hallford et al., 2020, 2023; Szpunar & Schacter, 2013). In investigating these factors, we explore whether the frequency of reading, which also requires active simulation of imagined hypothetical events, is a key factor in increasing cognitive flexibility and transportability, thereby enhancing future simulations.

We identify cognitive flexibility as a factor that enhances vividness of future simulations based on the *constructive episodic simulation hypothesis*, in which future simulations are thought to arise from the “flexible, constructive nature” of episodic memory (Schacter & Addis, 2007). To our knowledge, only two studies (Addis et al., 2016; Roberts et al., 2017) investigated how divergent thinking, assessed using the alternative uses task (Guilford, 1967), relates to EFT. Results of both studies showed that higher divergent thinking was associated with more detailed future simulations. Divergent thinking is one form of cognitive flexibility; however, it fails to capture multiple diverse aspects of this trait. In the present study, we administered the cognitive flexibility inventory (CFI; Dennis & Vander Wal, 2010), a multidimensional instrument designed to assess diverse facets of this construct. We examined whether cognitive flexibility measured as a trait variable is positively associated with vividness of future simulations, which, in turn, leads to a higher sense of perceived control over the future. When a future event feels more vivid, it leads to an increase in perceived control due to the increased familiarity and fluency of imagined events (Szpunar & Schacter, 2013) and a higher “belief in occurrence”—the subjective feeling surrounding whether a future event will unfold as imagined (D’Argembeau, 2025; see also Hallford et al., 2020, 2023).

In highlighting the importance of transportability for enhancing the vividness of future simulations, we turn to the literature on narrative transportation. Reading, like imagining a hypothetical future, requires the active construction of mental models to simulate events that are presented within a fictional narrative (Zwaan & Radvansky, 1998). *Narrative transportation* describes the phenomenon of becoming totally immersed or “transported” from the current world of origin into the alternate world of a story narrative (Gerrig, 1993). Therefore, the dispositional trait of transportability reflects that some individuals are more readily and deeply transported by narratives, thereby accruing more experience representing and simulating imagined events (Dal Cin et al., 2004). When in this state of immersion, we feel less urgency about our current world and our own goals are replaced by the goals of the protagonist (Cohen et al., 2015, 2023). In this way, when we become narratively transported, stories offer a portal into a cognitively flexible state. Individuals who are higher on transportability shift their beliefs and attitudes more flexibly in response to the narrative that they are consuming (Komori, 2018). Growing evidence suggests that the same brain regions that underlie reading fiction are also active when simulating hypothetical future events; notably, these brain regions are not activated for reading factual stories (Altmann et al., 2014). Thus, similar cognitive mechanisms are recruited whether simulating hypothetical future or imaginary fictional events.

Stories engage the reader because they are likely to trigger story-congruent personal thoughts and memories (Green & Appel, 2024). Therefore, while reading a fictional narrative, we include bits of autobiographical content that helps to fill out the story. Costabile (2016) suggests that the same mental models are employed whether we are reading a story or constructing our own personal narrative. The constructive nature of both future thinking and reading fiction involves a form of cognitive flexibility in the ability to fluidly shift and adapt cognitive processing to fit the narrative being experienced (while reading) and created (while future thinking). We propose that reading, especially reading in genres that prioritize engagement with complexity and difference, helps to hone the simulation capabilities that are later critical in flexibly and vividly simulating one’s own personal future. In a nationally representative, preregistered study, Buttrick et al. (2022) found that greater middle-school reading of young-adult, historical fiction, sci-fi/fantasy, and, especially, literary fiction predicted a more complex, flexible way of thinking about the social world, while increased middle-school romance-novel reading predicted a social worldview that was simpler, more essentialized, and more accepting of the status quo. As opposed to so-called genre fiction (such as romance or mystery novels), which more often privileges a reading experience of comfort, ease, and pleasure, Buttrick and colleagues suggest that some forms of fiction such as literary fiction are distinctive in how they push readers to alter their view of the world through the presentation of “difference” (see Barthes, 1975, for a similar distinction). They found that the reading patterns during middle-school were more predictive of present-day worldviews than reading in the current-day, implying that views about the social world are likely more malleable in childhood (Cheung et al., 2011). Furthermore, Gopnik and colleagues (2017) have demonstrated that during adolescence, there is a “second window” of flexibility that arises (the first window is in preschool years) where adolescents are more flexible problem solvers compared with adults.

In the present study, participants performed a detailed guided future simulation of a day 10 years in the future, after which they wrote a summary of their future simulation and rated its vividness and perceived control. We chose a temporal distance of 10 years based on findings that future thinking of temporally nearer events are typically more concrete and constrained by current practical concerns compared with more temporally distant events (D’Argembeau & Van der Linden, 2004; Trope & Liberman, 2003). The 10-year timeframe enabled participants to rely both on episodic memory and on *personal semantic memory* (Conway & Pleydell-Pearce, 2000), the fact-based knowledge a person has about their life that cannot be linked to a specific episode—a subcomponent of autobiographical knowledge that can inform one’s self concept (La Corte & Piolino, 2016). During simulations, specific probes were used based on the probes used in the episodic specificity induction by Schacter and Madore (2016) to encourage participants to imagine future simulations in more detail.

We predicted that cognitive flexibility and transportability would be positively associated with vividness of future simulations, which would lead to a higher sense of perceived control over the future. Furthermore, participants self-reported how often they read in several genres while in middle school along with their current reading habits. We hypothesized that increased reading of fiction in adolescence leads to increases in cognitive flexibility and transportability which, in turn, leads to higher vividness and perceived control of future simulations.

## Methods

### Participants

Our original sample included a convenience sample of 494 university-aged participants from an undergraduate research pool who volunteered to participate in this study for research participation credit (IRB 2025–0374—Visualizing Future Events). We removed 10 participants who failed to complete all the measures, resulting in a final sample size of 484 participants (366 women, 102 men, eight participants who chose not to disclose their gender). The mean age was 19.2 years (*SD* = 1.15). In the final sample, 330 participants identified as White, six as Black, 59 as Asian, and 37 as Hispanic. Forty-four participants chose either not to report their ethnicity or fell into an “Other” group. Two hundred and thirty-one participants had at least one parent with a graduate degree, 149 had parents with bachelor’s degrees, and 88 had parents with no college degrees. Eight participants chose not to disclose their parental education status. Simulations suggested that a sample of 475 would provide 80% power to detect a regression coefficient from reading to either cognitive flexibility or transportability in our structural equation model of *B* = 0.15.

### Materials and procedure

After obtaining informed consent, participants completed a questionnaire containing a set of measures and demographics. Some additional measures of personality traits were collected but not analyzed. The cognitive flexibility inventory (CFI) involved a 20-item self-report scale that measured three aspects of this construct including (a) the tendency to perceive difficult situations as controllable; (b) the ability to perceive multiple alternative explanations for life occurrences and human behavior; and (c) the ability to generate multiple alternative solutions to difficult situations (Dennis & Vander Wal, 2010). The CFI contained two Likert-type subscales (1 = s*trongly disagree* to 7 = *strongly agree*). The Alternatives subscale measured the ability to perceive multiple alternative explanations for difficult situations, life occurrences, and human behavior. An example question is, “I like to look at difficult situations from many different angles”; scale alpha = .91. The Control subscale measured the tendency to perceive difficult situations as controllable. An example question is, “I am capable of overcoming the difficulties in life that I face”; scale alpha = .86. The transportability scale included 13 items measuring transportability, the degree to which individuals typically become immersed in a story (Jensen et al., 2016). An example item is, “I sometimes feel as if I am part of the story” (1 = *strongly disagree* to 5 = *strongly agree*); scale alpha = 0.89.

Participants were asked to report on their reading both during middle school as well as their current reading habits, using a prompt “How often do/did you read the following sorts of books? If a book crosses genre boundaries, go ahead and count it in both.” Genres were drawn from the most popular genres listed on the book review social network Goodreads, and included Academic, Biography, Comedy, Cookbooks, Essays/Criticism, Health, History, Memoir, Self-Help, Spirituality, Travel, and Other Nonfiction (i.e., nonfiction not listed above), all grouped into a Non-Fiction category); as well as and Crime/Mystery/Suspense, Fantasy, Graphic Novels/Comics/Manga, Historical Fiction, Horror, Literary Fiction, Poetry, Romance, Science Fiction, Urban, Young Adult, and Other Fiction (i.e., fiction not listed above), all grouped into a Fiction category. Participants could respond with the following options for each genre: Never, Rarely, Occasionally, Sometimes, Often, Very Often, and Constantly. The same question was asked about current reading. Descriptives for these items can be found in the online supplement.

For the guided simulation of an average day 10 years in the future, probes were based on the episodic specificity induction used by Schacter and Madore (2016). We chose a temporal distance of 10 years based on findings that future thinking of temporally nearer events can be more constrained by current practical concerns compared with more temporally distant events (D’Argembeau & Van der Linden, 2004; Trope & Liberman, 2003). Our choice to use specific time-of-day probes was intended to provide a type of temporal and thematic structure that would anchor future simulations to a familiar routine thereby providing consistency across participants. Participants were asked to close their eyes while experimenters led them through several probes to help them to imagine what they might be doing on this day. They were encouraged to simulate events in chronological order from morning to evening:

*–Morning–*

*Imagine yourself waking up in the morning. What does your bedroom look like? What type of clothing will you wear on this day? Does your hair look different 10 years later? What will you do for breakfast? Will you eat at home or grab something on your way to work. What work are you doing? Who will you be working with?*

*[Close your eyes and imagine it for a few minutes]*

*–Afternoon–*

*Maybe you are working on an exciting new project. What is the project? Are you working on your own or with colleagues? Who are your colleagues? What do you see around you? Who is in the room with you?*

*[Close your eyes and imagine it for a few minutes]*

*–Evening–*

*After you finish work for the day, what will you do? Will you go home or meet friends or your spouse for dinner? Maybe you will go to the gym? Perhaps you will go to a pub or see a new movie? If you live near a park or beach, maybe you will take your dog for a walk. Eventually, you return home to wind down before you go to sleep.*

*[Close your eyes and imagine it for a few minutes]*

After the guided simulation, participants wrote a summary of their simulation. Episodic future thinking was measured by questions that measured self-reported vividness, “How vivid and detailed are your thoughts of this future event?”; alpha across the morning, afternoon, and evening visualizations = 0.84; as well as perceived control, “How much control do you feel to be able to make this future event happen in the way you imagine it?”; alpha across the morning, afternoon, and evening visualizations = 0.86. Participants responded using a 10-point scale (0 = *not at all* to 10 = *extremely*). Questions measuring vividness and perceived control were adapted from Hallford et al. (2020).

## Results

An alpha level of .05 was used for all analyses unless otherwise noted. We conducted preliminary analyses to examine the influence of trait cognitive flexibility and transportability on episodic future thinking while controlling for current-day fiction reading. We first predicted the vividness of future simulations jointly from both trait cognitive flexibility and transportability, controlling for present-day fiction reading. The two predictors significantly explained 19.1% of the variance in vividness of future simulations, *R*^2^ = .191, *F*(3, 476) = 37.58, *p* < .001, Cohen’s *f*^2^ = .24. Both cognitive flexibility (*β* = .19, *p* < .001) and transportability (*β* = .36, *p* < .001) significantly predicted vividness of future thinking. The amount of present-day reading was not a significant predictor of vividness, *t*(476) = − .30, *p* = .77.

We next predicted perceived control of future simulations from the same two predictors and controls. The two predictors significantly explained 11.1% of the variance in the perceived control of future simulations, *R*^2^ = .111, *F*(3, 476) = 19.81, *p* < .001, Cohen’s *f*^2^ = .12. Cognitive flexibility (*β* = .25, *p* < .001) significantly predicted perceived control of the future as did transportability (*β* = .17, *p* < .001). The amount of present-day reading was not a significant predictor of perceived control, *t*(476) = .09, *p* = .93 (see Table S1 for descriptives and correlations for all variables).

### Aggregated measures of reading

**Fig. 1:**
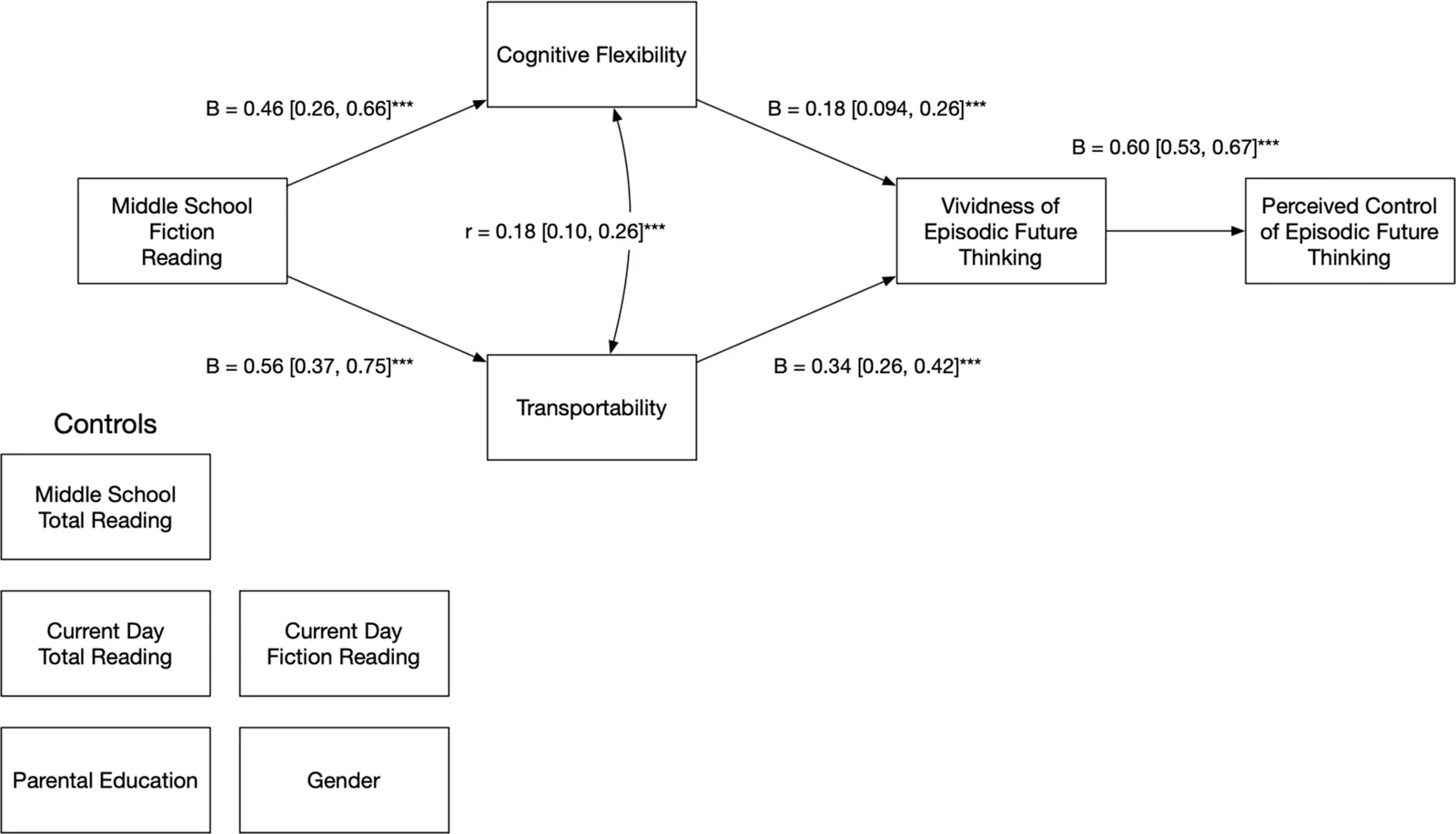
Path diagram for the structural equation model using middle-school fiction reading as a predictor. ***p < .001

We next built a set of structural equation models to understand the relationship between reading, cognitive flexibility, transportability, vividness, and perceived control of future simulations. We created four distinct models to understand the separate relationships with our psychological variables of 1) middle-school fiction reading, 2) middle-school nonfiction reading, 3) present-day fiction reading, and 4) present-day nonfiction reading. We created reading composites, averaged across the types of reading measured (see Table S2 for SEM parameters for aggregate reading measures). All scales were scored as per guidelines, and all measures were entered as standardized manifest variables. Means and variances were allowed to freely vary for all items. For our models of middle-school reading, we additionally controlled for present-day reading of the same sort (i.e., controlling for present-day fiction reading when modeling the effect of middle-school fiction reading; see Fig. 1 for a schematic of the paths).

The four models each showed evidence of good fit: middle-school fiction—AIC = 11,338.80, BIC = 11,378.34, CFI = 0.987, TLI = 0.958, RMSEA = 0.069 [0.043, 0.095]; middle-school nonfiction—AIC = 11,431.43, BIC = 11,470.96, CFI = 0.987, TLI = 0.957, RMSEA = 0.069 [0.043, 0.095]; present-day fiction—AIC = 10,813.55, BIC = 10,848.02, CFI = 0.98, TLI = 0.94, RMSEA = 0.076 [0.049, 0.11]; present-day nonfiction—AIC = 10,891.33, BIC = 10,925.79, CFI = 0.98, TLI = 0.93, RMSEA = 0.076 [0.049, 0.11].

Controlling for total self-reported middle-school reading, total current-day reading, total current fiction reading, gender, and parental education, we found that those who read more fiction in middle school reported higher degrees of both cognitive flexibility and transportability, and that both higher degrees of cognitive flexibility and transportability predicted increased vividness of their future life, which, in turn, predicted a greater sense that their future life was controllable (see Fig. 1 for the full path diagram and all parameter estimates).

**Fig. 2:**
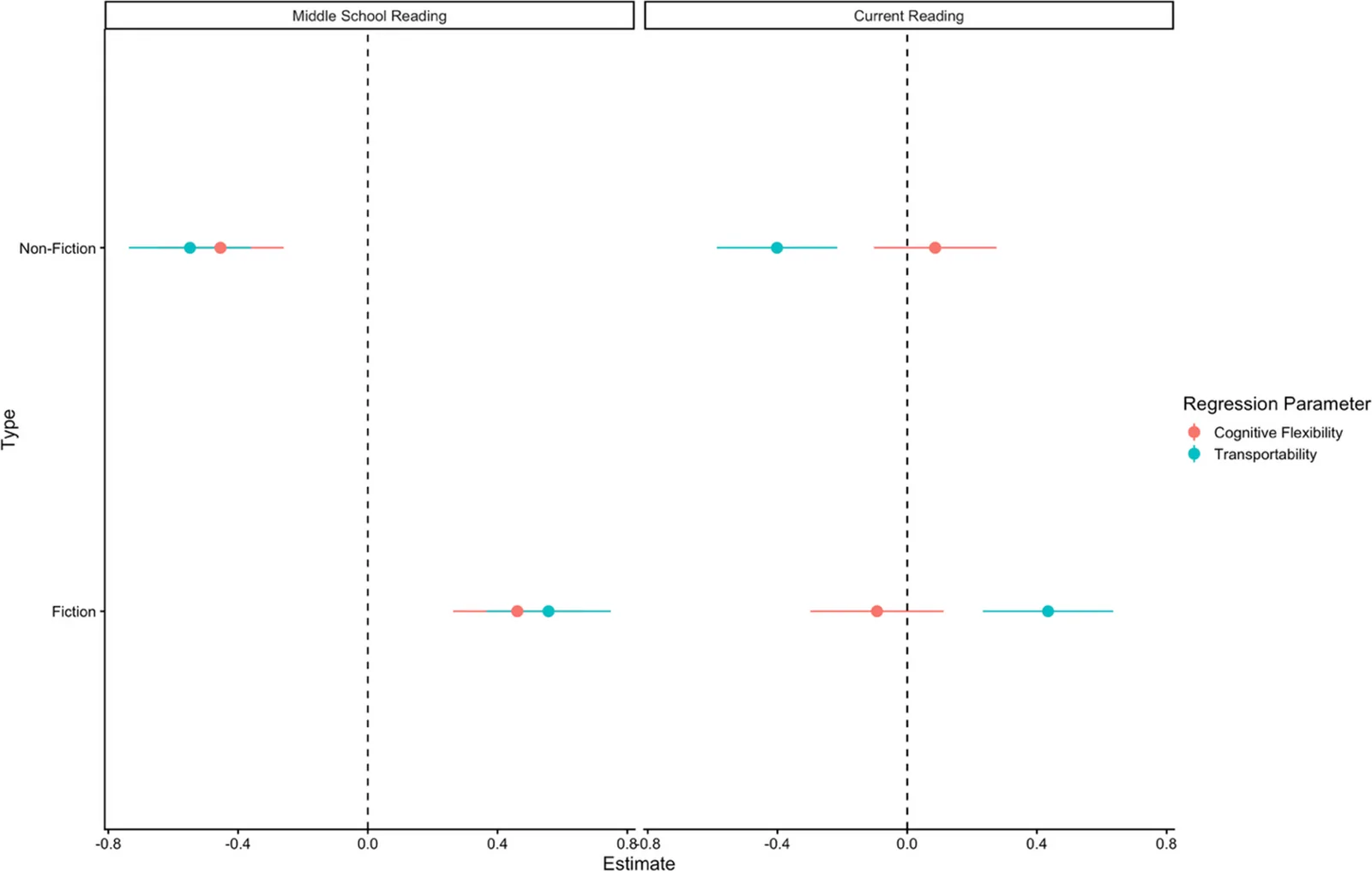
Standardized regression parameters for the relationship between aggregated reading indices and both cognitive flexibility and transportability extracted from separate structural equation models modeling middle school fiction, middle-school nonfiction, current-day fiction, and current-day nonfiction reading. Error bars indicate 95% confidence intervals. (Color figure online)

We found mixed evidence for a relationship between middle-school nonfiction reading or present-day reading on our psychological variables. We present the parameters of the key regressions from type of reading to both cognitive flexibility and transportability in Fig. 2. The parameters for the rest of the structural equation models can be found in the online supplement—as our sample does not meaningfully change from model to model, the parameters for the elements of the model that do not include reading (and that therefore do not vary across models) are essentially unchanged. We find that only middle-school fiction reading is significantly associated with both increased cognitive flexibility and transportability. Middle-school nonfiction reading is associated with reduced present-day cognitive flexibility, *B* = − 0.45 [− 0.65, − 0.26], *p* < .001 and reduced transportability, *B* = − 0.55 [− 0.74, − 0.36], *p* < .001. Present-day fiction reading was significantly associated with increased transportability, *B* = 0.43 [0.23, 0.64], *p* < .001, but we found no evidence for a relationship with cognitive flexibility, *B* = − 0.093 [− 0.30, 0.11], *p* = .37. Present-day nonfiction was associated with reduced transportability, *B* = − 0.40 [− 0.59, − 0.21]*, p* < .001, but we found no evidence for a relationship with cognitive flexibility, *B* = 0.086 [− 0.10, 0.28], *p* = .37 (see Fig. S1 for alternate path models and Table S3 for fit indices in the online supplement).

### Disaggregated measures of reading

**Fig. 3:**
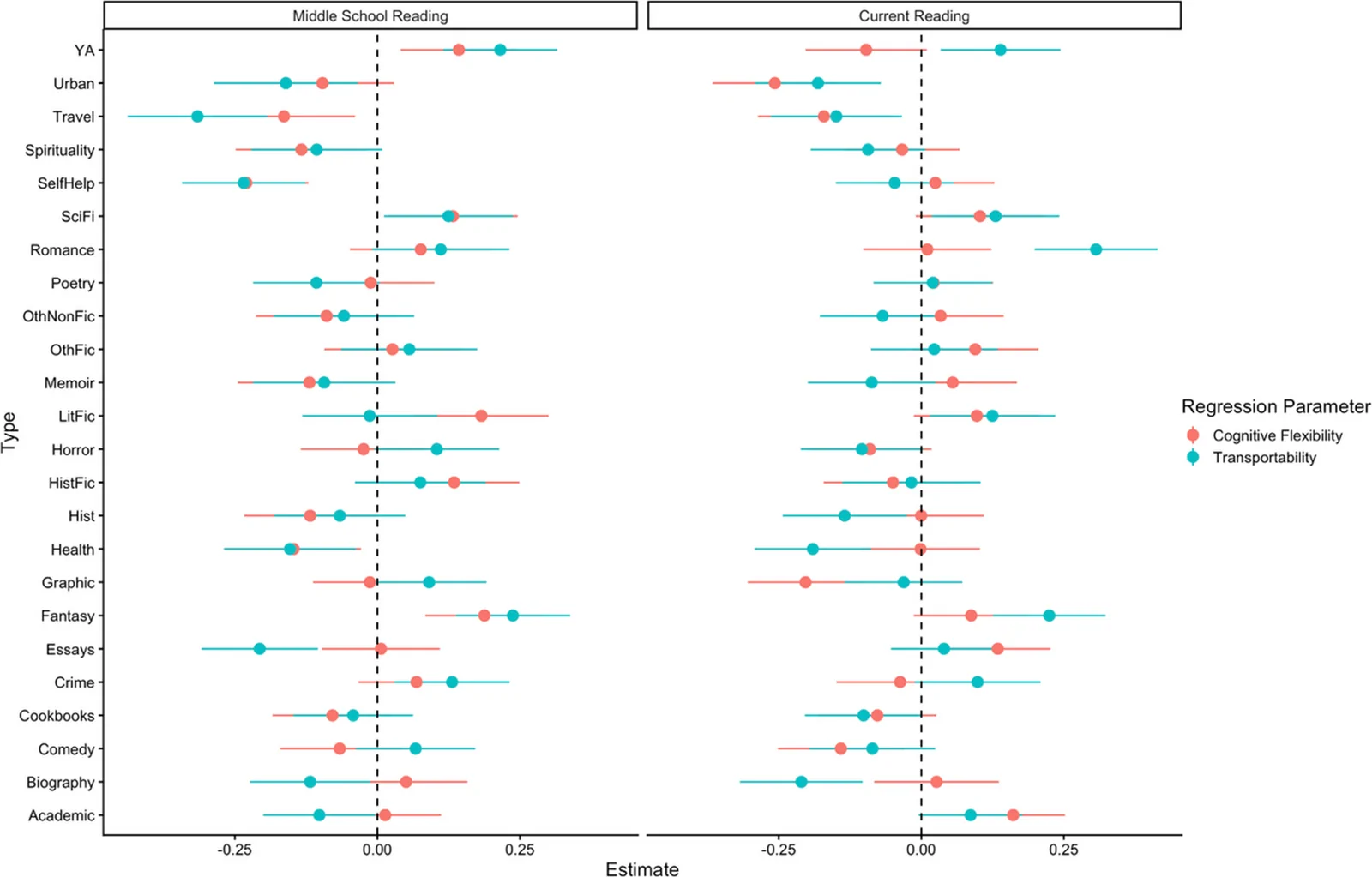
Standardized regression parameters for the relationship between disaggregated reading indices and both cognitive flexibility and transportability extracted from 48 separate structural equation models investigating the effects of particular genres and reading windows. Error bars indicate 95% confidence intervals. (Color figure online)

We then disaggregated our genres of reading to examine whether there were distinct patterns across the precise types of reading our participants engaged in, building separate models for each combination of reading genre and timeframe of reading (i.e., 24 genres × middle-school/contemporary reading = 48 separate models). Models followed the same form as our aggregated models, controlling for total self-reported middle-school reading, total current-day reading, gender, and parental education. For our 24 middle-school-reading models, we additionally controlled for the equivalent volume of reading in that genre in the present day (i.e., for modeling middle-school literary fiction reading, we additionally controlled for current-day literary fiction reading). As above, since the parts of the model that do not directly involve our reading measures do not meaningfully change across models, we present only the regression pathways from type of reading to both cognitive flexibility and transportability. All parameters, however, can be found in the online supplement for interested readers. See the key regression parameters for all models in Fig. 3.

Figure 3 shows that only those forms of reading that ask young readers to encounter minds and worlds different from their own were associated with greater degrees of current-day cognitive flexibility: middle-school literary-fiction reading (*B* = 0.18 [0.065, 0.30], *p* = .002), middle-school young adult (*B* = 0.14 [0.041, 0.24], *p* = .006), middle-school science fiction (*B* = 0.13 [0.019, 0.25], *p* = .021), middle-school historical fiction (*B* = 0.14 [0.020, 0.25, *p* = .020), and middle-school fantasy (*B* = 0.19 [0.084, 0.29], *p* < .001). When looking at present-day reading habits, cognitive flexibility was only positively associated with increased reading of essays (*B* = 0.13 [0.042, 0.23], *p* = .0042) and academic texts (*B* = 0.16 [0.070, 0.25], *p* < .001).

Increased transportability was associated with increased reading, in middle school, of young-adult fiction (*B* = 0.22 [0.12, 0.32], *p* < .001), sci-fi (*B* = 0.13 [0.012, 0.24], *p* = .030), fantasy (*B* = 0.24 [0.14, 0.34], *p* < .001), and crime (*B* = 0.13 [0.030, 0.23], *p* = .010); and with increased reading, in adulthood, of literary fiction (*B* = 0.13 [0.015, 0.24], *p* = .025), young adult fiction (*B* = 0.14 [0.034, 0.24], *p* = .0091), science fiction (*B* = 0.13 [0.018, 0.24], *p* = .022), romance (*B* = 0.31 [0.20, 0.41], *p* < .001), and fantasy (*B* = 0.22 [0.13, 0.32], *p* < .001).

All parameter estimates can be found in the online supplement.

## Discussion

To our knowledge, the current study is the first to identify two valid and reliable traits, cognitive flexibility and transportability, that are positively associated with higher vividness and perceived control of the future. We found that greater reading of fiction in middle school, especially the sorts of reading that ask readers to grapple with disparate and unpredictable worlds, predicted higher degrees of both cognitive flexibility and transportability in the present day.

Our measure of cognitive flexibility as a stable trait replicates and extends prior work that demonstrated a positive association between divergent thinking and number of details in future simulations (Addis et al., 2016; Roberts et al., 2017). Our results demonstrate that the benefit of cognitive flexibility is observed not only in number of details in simulations but also in the vividness and perceived control of future simulations. While cognitive flexibility emerged as a reliable predictor of vividness of future thought, it had an especially robust association with perceived control of future simulations. Given that our index of cognitive flexibility measures the ability to generate multiple explanations for human behavior, multiple solutions to problems, and to perceive difficult problems as solvable, it makes sense that this variable was especially important in predicting a sense of control over the future.

Transportability was positively associated with both vividness and perceived control of the future, with an especially large association with vividness of future thinking. Our results suggest that greater immersion and practice with being transported into fictional worlds enhances the vividness of our own simulations of the future. This supports the episodic simulation hypothesis, which posits that future thinking draws upon and recombines details from past experiences, in this case, past fictional narratives, to build future scenarios (Schacter & Addis, 2007).

“Scene construction,” the ability to mentally generate and maintain a coherent, multimodal spatial representation (Hassabis & Maguire, 2007) is conceptually similar to the notion of “situation models,” mental models created to represent fictional events and characters (Zwaan & Radvansky, 1998). Cognitive flexibility and transportability may serve as “tools” that aid with disengagement from the present world to construct and generate representations of a different one. Increased reading in early adolescence may hone these abilities.

Importantly, not all types of reading, and not even all types of fiction, have the same predictive effects. As in Buttrick et al. (2022), early-life reading of literary fiction, young-adult fiction, sci-fi, fantasy, and historical fiction had distinctive effects on future worldviews. We find that reading more in genres that regularly ask readers to step into unfamiliar worlds is associated with increased cognitive flexibility, much like prior work found that reading in those specific genres were associated with increased flexibility in making sense of the social world. When it comes to the reading of nonfiction, we find no evidence for positive aggregated effects, but there are some scattered relationships with reading of essays and academics in the present day, perhaps reflecting the educated nature of our sample. Reading seems to be most impactful in one’s early life, when worldviews are still being formed (see Buttrick et al., 2022; Lenhart et al., 2023, for similar findings).

Several limitations should be considered when drawing conclusions from our data. Our sample is relatively young and not a representative one, and so care must be taken in generalizing it to other Americans. We also rely on self-report in our reading measures, which means we are reliant on participants’ recollection of their middle-school reading habits (though see Buttrick et al., 2022, for previous use of retrospective self-report of reading habits and a preregistered replication of a university-based sample using a middle-aged, nationally representative sample). Finally, our results are correlational. While we attempt to statistically control for a set of plausible third variables (parental education, overall reading volume, and gender), it is plausible that other variables may explain middle-school reading habits and our psychological variables. Future work that builds upon our findings while controlling for these potential limiting factors will be important.

A possible alternative interpretation of our findings is that those who rate highly on traits such as cognitive flexibility and transportability would be drawn to certain sorts of reading. However, one would then expect that present-day reading would be more strongly associated with these traits, especially since participants would have greater freedom in choosing what to read in the present day than when they were in middle school. Instead, we find that middle-school reading is more strongly associated with flexibility and transportability—a finding more in line with a model in which reading helps readers to develop these qualities. We additionally find that our measures of middle-school reading continue to predict flexibility and transportability above and beyond present-day reading habits in the same genres, further strengthening our case that middle-school reading specifically is an important element in the development of episodic future thinking.

Other forms of imaginative play may have similar outcomes. In early childhood, pretend play is linked with increases in counterfactual reasoning (Buchsbaum et al., 2012) and cognitive flexibility (Russ & Vargo, 2025). We suggest that mentally representing imaginary scenarios that are different and untethered to the immediate external world are the drivers of processes such as cognitive flexibility and transportability that enhance future simulation capabilities. Future work investigating whether fictional narratives that are presented through other mediums, such as video, which visually scaffolds some of the mental world-building that reading forces a reader to do on their own, helps to develop these same skills.

In sum, the current results add to the body of knowledge on episodic future thinking by identifying cognitive flexibility and transportability as traits that contribute to higher vividness and a higher subjective feeling of control over the future, with greater reading of fiction in adolescence as a factor that predicted higher levels of these traits. The efficacy with which we can imagine the future has powerful consequences for feelings surrounding the reliability of the future (D’Argembeau, 2025). Therefore, the more vivid and coherent a future simulation, the stronger the belief that it could occur, thereby making long-term goals feel more concrete and achievable—which may increase motivation to take necessary steps in the present.

## Supplementary Information

Below is the link to the electronic supplementary material.

Supplementary file1 (DOCX 306 kb)

## Data Availability

To maximize transparency and reproducibility, the full text of the materials, along with all data and analysis scripts including participants’ free-response descriptions of their future life simulations can be found at: https://osf.io/fdhvz/overview?view_only=36d8b0a8354b4b61b7d974a1e2783e9b
