# Supplementary material for: Reading fiction in early adolescence predicts the ability to imagine our own future
Source: Psychon Bull Rev. 2026 Jul 29;33(6):198. doi: 10.3758/s13423-026-02973-w (PMC13421237; doi:10.3758/s13423-026-02973-w)
Supplement: Supplementary file 1 — Supplementary file1 (DOCX 306 kb) [file 13423_2026_2973_MOESM1_ESM.docx]

Table S1

*Descriptives and correlations for all variables*

| Variable | *M* | *SD* | 1 | 2 | 3 |
| --- | --- | --- | --- | --- | --- |
|  |  |  |  |  |  |
| 1. Cognitive Flexibility | 5.00 | 0.65 |  |  |  |
|  |  |  |  |  |  |
| 2. Transportability | 3.79 | 0.64 | .20*** |  |  |
|  |  |  | [.12, .29] |  |  |
|  |  |  |  |  |  |
| 3. Vividness | 8.19 | 1.69 | .25*** | .38*** |  |
|  |  |  | [.16, .33] | [.30, .45] |  |
|  |  |  |  |  |  |
| 4. Controllability | 8.06 | 1.76 | .32*** | .22*** | .60*** |
|  |  |  | [.24, .40] | [.13, .30] | [.54, .66] |
|  |  |  |  |  |  |

*Note.* Values in square brackets indicate the 95% confidence interval for each correlation. **p* < .05, ***p* < .01, *** *p* < .001

Table S2

*SEM parameters for aggregate reading measures*

| Parameter | Estimate | 95% CI | *p* |  |
| --- | --- | --- | --- | --- |
| Middle School Fiction | | | | |
| Reading -> Cognitive Flexibility | 0.46 | [0.26, 0.66] | <.001 | *** |
| Reading -> Transportability | 0.56 | [0.37, 0.75] | <.001 | *** |
| Cognitive Flexibility <-> Transportability | 0.18 | [0.10, 0.26] | <.001 | *** |
| Cognitive Flexibility -> Vividness | 0.18 | [0.094, 0.26] | <.001 | *** |
| Transportability -> Vividness | 0.34 | [0.26, 0.42] | <.001 | *** |
| Vividness -> Controllability | 0.60 | [0.53, 0.67] | <.001 | *** |
| Middle School NonFiction | | | | |
| Reading -> Cognitive Flexibility | −0.45 | [−0.65, −0.26] | <.001 | *** |
| Reading -> Transportability | −0.55 | [−0.74, −0.36] | <.001 | *** |
| Cognitive Flexibility <-> Transportability | 0.18 | [0.10, 0.26] | <.001 | *** |
| Cognitive Flexibility -> Vividness | 0.18 | [0.094, 0.26] | <.001 | *** |
| Transportability -> Vividness | 0.34 | [0.26, 0.42] | <.001 | *** |
| Vividness -> Controllability | 0.60 | [0.53, 0.67] | <.001 | *** |
| Current Fiction | | | | |
| Reading -> Cognitive Flexibility | −0.09 | [−0.30, 0.11] | .37 |  |
| Reading -> Transportability | 0.43 | [0.23, 0.64] | <.001 | *** |
| Cognitive Flexibility <-> Transportability | 0.22 | [0.14, 0.31] | <.001 | *** |
| Cognitive Flexibility -> Vividness | 0.18 | [0.09, 0.26] | <.001 | *** |
| Transportability -> Vividness | 0.34 | [0.26, 0.42] | <.001 | *** |
| Vividness -> Controllability | 0.60 | [0.53, 0.67] | <.001 | *** |
| Current NonFiction | | | | |
| Reading -> Cognitive Flexibility | 0.09 | [−0.10, 0.28] | .37 |  |
| Reading -> Transportability | −0.40 | [−0.59, −0.22] | <.001 | *** |
| Cognitive Flexibility <-> Transportability | 0.22 | [0.14, 0.31] | <.001 | *** |
| Cognitive Flexibility -> Vividness | 0.18 | [0.09, 0.26] | <.001 | *** |
| Transportability -> Vividness | 0.34 | [0.26, 0.42] | <.001 | *** |
| Vividness -> Controllability | 0.60 | [0.53, 0.67] | <.001 | *** |

Table S3

*SEM parameters for genre reading measures*

| Parameter | Estimate | 95% CI | *p* |  |
| --- | --- | --- | --- | --- |
| Middle School Academic | | | | |
| Reading -> Cognitive Flexibility | 0.014 | [−0.084, 0.11] | .78 |  |
| Reading -> Transportability | −0.10 | [−0.20, −0.004] | .040 | * |
| Cognitive Flexibility <-> Transportability | 0.21 | [0.13, 0.293] | <.001 | *** |
| Cognitive Flexibility -> Vividness | 0.18 | [0.094, 0.26] | <.001 | *** |
| Transportability -> Vividness | 0.34 | [0.26, 0.42] | <.001 | *** |
| Vividness -> Controllability | 0.60 | [0.53, 0.67] | <.001 |  |
| Middle School Biography | | | | |
| Reading -> Cognitive Flexibility | 0.050 | [−0.057, 0.16] | .36 |  |
| Reading -> Transportability | −0.12 | [−0.22, −0.013] | .027 | * |
| Cognitive Flexibility <-> Transportability | 0.22 | [0.14, 0.31] | <.001 | *** |
| Cognitive Flexibility -> Vividness | 0.18 | [0.094, 0.26] | <.001 | *** |
| Transportability -> Vividness | 0.34 | [0.26, 0.42] | <.001 | *** |
| Vividness -> Controllability | 0.60 | [0.53, 0.67] | <.001 | *** |
| Middle School Comedy | | | | |
| Reading -> Cognitive Flexibility | −0.066 | [−0.17, 0.039] | .22 |  |
| Reading -> Transportability | 0.067 | [−0.038, 0.17] | .21 |  |
| Cognitive Flexibility <-> Transportability | 0.21 | [0.13, 0.30] | <.001 | *** |
| Cognitive Flexibility -> Vividness | 0.18 | [0.094, 0.26] | <.001 | *** |
| Transportability -> Vividness | 0.34 | [0.26, 0.42] | <.001 | *** |
| Vividness -> Controllability | 0.60 | [0.53, 0.67] | <.001 | *** |
| Middle School Cookbooks | | | | |
| Reading -> Cognitive Flexibility | −0.079 | [−0.18, 0.027] | .14 |  |
| Reading -> Transportability | −0.043 | [−0.15, 0.063] | .42 |  |
| Cognitive Flexibility <-> Transportability | 0.21 | [0.19, 0.30] | <.001 | *** |
| Cognitive Flexibility -> Vividness | 0.18 | [0.094, 0.26] | <.001 | *** |
| Transportability -> Vividness | 0.34 | [0.26, 0.42] | <.001 | *** |
| Vividness -> Controllability | 0.60 | [0.53, 0.67] | <.001 | *** |
| Middle School Crime | | | | |
| Reading -> Cognitive Flexibility | 0.069 | [−0.033, 0.17] | .18 |  |
| Reading -> Transportability | 0.13 | [0.030, 0.23] | .010 | * |
| Cognitive Flexibility <-> Transportability | 0.21 | [0.13, 0.30] | <.001 | *** |
| Cognitive Flexibility -> Vividness | 0.18 | [0.094, 0.26] | <.001 | *** |
| Transportability -> Vividness | 0.34 | [0.26, 0.42] | <.001 | *** |
| Vividness -> Controllability | 0.60 | [0.53, 0.67] | <.001 | *** |
| Middle School Essays | | | | |
| Reading -> Cognitive Flexibility | 0.006 | [−0.097, 0.11] | .91 |  |
| Reading -> Transportability | −0.21 | [−0.31, −0.10] | <.001 | *** |
| Cognitive Flexibility <-> Transportability | 0.21 | [0.13, 0.30] | <.001 | *** |
| Cognitive Flexibility -> Vividness | 0.18 | [0.094, 0.26] | <.001 | *** |
| Transportability -> Vividness | 0.34 | [0.26, 0.42] | <.001 | *** |
| Vividness -> Controllability | 0.60 | [0.53, 0.67] | <.001 | *** |
| Middle School Fantasy | | | | |
| Reading -> Cognitive Flexibility | 0.19 | [0.084, 0.29] | <.001 | *** |
| Reading -> Transportability | 0.24 | [0.14, 0.34] | <.001 | *** |
| Cognitive Flexibility <-> Transportability | 0.17 | [0.096, 0.26] | <.001 | *** |
| Cognitive Flexibility -> Vividness | 0.18 | [0.094, 0.26] | <.001 | *** |
| Transportability -> Vividness | 0.34 | [0.26, 0.42] | <.001 | *** |
| Vividness -> Controllability | 0.60 | [0.53, 0.67] | <.001 | *** |
| Middle School Graphic | | | | |
| Reading -> Cognitive Flexibility | −0.013 | [−0.11, 0.087] | .79 |  |
| Reading -> Transportability | 0.091 | [−0.010, 0.19] | .074 |  |
| Cognitive Flexibility <-> Transportability | 0.21 | [0.13, 0.30] | <.001 | *** |
| Cognitive Flexibility -> Vividness | 0.18 | [0.094, 0.26] | <.001 | *** |
| Transportability -> Vividness | 0.34 | [0.26, 0.42] | <.001 | *** |
| Vividness -> Controllability | 0.60 | [0.53, 0.67] | <.001 | *** |
| Middle School Health | | | | |
| Reading -> Cognitive Flexibility | −0.15 | [−0.27, −0.029] | .014 | * |
| Reading -> Transportability | −0.15 | [−0.27, −0.037] | .009 | ** |
| Cognitive Flexibility <-> Transportability | 0.21 | [0.13, 0.29] | <.001 | *** |
| Cognitive Flexibility -> Vividness | 0.18 | [0.094, 0.26] | <.001 | *** |
| Transportability -> Vividness | 0.34 | [0.26, 0.42] | <.001 | *** |
| Vividness -> Controllability | 0.60 | [0.53, 0.67] | <.001 | *** |
| Middle School HistFic | | | | |
| Reading -> Cognitive Flexibility | 0.14 | [0.020, 0.25] | .020 | * |
| Reading -> Transportability | 0.075 | [−0.039, 0.19] | .19 |  |
| Cognitive Flexibility <-> Transportability | 0.21 | [0.13, 0.30] | <.001 | *** |
| Cognitive Flexibility -> Vividness | 0.18 | [0.094, 0.26] | <.001 | *** |
| Transportability -> Vividness | 0.34 | [0.26, 0.42] | <.001 | *** |
| Vividness -> Controllability | 0.60 | [0.53, 0.67] | <.001 | *** |
| Middle School Hist | | | | |
| Reading -> Cognitive Flexibility | −0.12 | [−0.234 −0.002] | .044 | * |
| Reading -> Transportability | −0.066 | [−0.18, 0.049] | .258 |  |
| Cognitive Flexibility <-> Transportability | 0.21 | [0.13, 0.30] | <.001 | *** |
| Cognitive Flexibility -> Vividness | 0.17 | [0.094, 0.26] | <.001 | *** |
| Transportability -> Vividness | 0.34 | [0.26, 0.42] | <.001 | *** |
| Vividness -> Controllability | 0.60 | [0.53, 0.67] | <.001 | *** |
| Middle School Horror | | | | |
| Reading -> Cognitive Flexibility | −0.024 | [−0.13, 0.086] | .66 |  |
| Reading -> Transportability | 0.10 | [−0.005, 0.21] | .059 |  |
| Cognitive Flexibility <-> Transportability | 0.21 | [0.13, 0.30] | <.001 | *** |
| Cognitive Flexibility -> Vividness | 0.18 | [0.094, 0.26] | <.001 | *** |
| Transportability -> Vividness | 0.34 | [0.26, 0.42] | <.001 | *** |
| Vividness -> Controllability | 0.60 | [0.53, 0.67] | <.001 | *** |
| Middle School LitFic | | | | |
| Reading -> Cognitive Flexibility | 0.18 | [0.065, 0.30] | .002 | ** |
| Reading -> Transportability | −0.013 | [−0.13, 0.11] | .82 |  |
| Cognitive Flexibility <-> Transportability | 0.21 | [0.13, 0.30] | <.001 | *** |
| Cognitive Flexibility -> Vividness | 0.18 | [0.094, 0.26] | <.001 | *** |
| Transportability -> Vividness | 0.34 | [0.26, 0.42] | <.001 | *** |
| Vividness -> Controllability | 0.60 | [0.53, 0.67] | <.001 | *** |
| Middle School Memoir | | | | |
| Reading -> Cognitive Flexibility | −0.12 | [−0.25, 0.006] | .061 |  |
| Reading -> Transportability | −0.093 | [−0.22, 0.032] | .140 |  |
| Cognitive Flexibility <-> Transportability | 0.22 | [0.13, 0.30] | <.001 | *** |
| Cognitive Flexibility -> Vividness | 0.18 | [0.094, 0.26] | <.001 | *** |
| Transportability -> Vividness | 0.34 | [0.26, 0.42] | <.001 | *** |
| Vividness -> Controllability | 0.60 | [0.53, 0.67] | <.001 | *** |
| Middle School Poetry | | | | |
| Reading -> Cognitive Flexibility | −0.012 | [−0.12, 0.10] | .84 |  |
| Reading -> Transportability | −0.11 | [−0.22, 0.004] | .057 |  |
| Cognitive Flexibility <-> Transportability | 0.22 | [0.14, 0.31] | <.001 | *** |
| Cognitive Flexibility -> Vividness | 0.18 | [0.094, 0.26] | <.001 | *** |
| Transportability -> Vividness | 0.34 | [0.26, 0.42] | <.001 | *** |
| Vividness -> Controllability | 0.60 | [0.53, 0.67] | <.001 | *** |
| Middle School Romance | | | | |
| Reading -> Cognitive Flexibility | 0.076 | [−0.048, 0.20] | .230 |  |
| Reading -> Transportability | 0.11 | [−0.009, 0.23] | .068 |  |
| Cognitive Flexibility <-> Transportability | 0.21 | [0.13, 0.30] | <.001 | *** |
| Cognitive Flexibility -> Vividness | 0.18 | [0.094, 0.26] | <.001 | *** |
| Transportability -> Vividness | 0.34 | [0.26, 0.42] | <.001 | *** |
| Vividness -> Controllability | 0.60 | [0.53, 0.67] | <.001 | *** |
| Middle School SciFi | | | | |
| Reading -> Cognitive Flexibility | 0.13 | [0.019, 0.25] | .021 | * |
| Reading -> Transportability | 0.13 | [0.012, 0.24] | .030 | * |
| Cognitive Flexibility <-> Transportability | 0.20 | [0.12, 0.29] | <.001 | *** |
| Cognitive Flexibility -> Vividness | 0.18 | [0.094, 0.26] | <.001 | *** |
| Transportability -> Vividness | 0.34 | [0.26, 0.42] | <.001 | *** |
| Vividness -> Controllability | 0.60 | [0.53, 0.67] | <.001 | *** |
| Middle School SelfHelp | | | | |
| Reading -> Cognitive Flexibility | −0.23 | [−0.34, −0.12] | <.001 | *** |
| Reading -> Transportability | −0.23 | [−0.34, −0.13] | <.001 | *** |
| Cognitive Flexibility <-> Transportability | 0.19 | [0.11, 0.27] | <.001 | *** |
| Cognitive Flexibility -> Vividness | 0.18 | [0.094, 0.26] | <.001 | *** |
| Transportability -> Vividness | 0.34 | [0.26, 0.42] | <.001 | *** |
| Vividness -> Controllability | 0.60 | [0.53, 0.67] | <.001 | *** |
| Middle School Spirituality | | | | |
| Reading -> Cognitive Flexibility | −0.13 | [−0.25, −0.018] | .023 | * |
| Reading -> Transportability | −0.11 | [−0.22, 0.009] | .067 |  |
| Cognitive Flexibility <-> Transportability | 0.21 | [0.13, 0.29] | <.001 | *** |
| Cognitive Flexibility -> Vividness | 0.18 | [0.094, 0.26] | <.001 | *** |
| Transportability -> Vividness | 0.34 | [0.26, 0.42] | <.001 | *** |
| Vividness -> Controllability | 0.60 | [0.53, 0.67] | <.001 | *** |
| Middle School Travel | | | | |
| Reading -> Cognitive Flexibility | −0.16 | [−0.29, −0.039] | .010 | ** |
| Reading -> Transportability | −0.32 | [−0.44, −0.19] | <.001 | *** |
| Cognitive Flexibility <-> Transportability | 0.18 | [0.10, 0.26] | <.001 | *** |
| Cognitive Flexibility -> Vividness | 0.18 | [0.094, 0.26] | <.001 | *** |
| Transportability -> Vividness | 0.34 | [0.26, 0.42] | <.001 | *** |
| Vividness -> Controllability | 0.60 | [0.53, 0.67] | <.001 | *** |
| Middle School Urban | | | | |
| Reading -> Cognitive Flexibility | −0.096 | [−0.22, 0.029] | .13 |  |
| Reading -> Transportability | −0.16 | [−0.29, −0.035] | .012 | * |
| Cognitive Flexibility <-> Transportability | 0.18 | [0.10, 0.27] | <.001 | *** |
| Cognitive Flexibility -> Vividness | 0.18 | [0.094, 0.26] | <.001 | *** |
| Transportability -> Vividness | 0.34 | [0.26, 0.42] | <.001 | *** |
| Vividness -> Controllability | 0.60 | [0.53, 0.67] | <.001 | *** |
| Middle School YA | | | | |
| Reading -> Cognitive Flexibility | 0.14 | [0.041, 0.25] | .006 | ** |
| Reading -> Transportability | 0.22 | [0.12, 0.32] | <.001 | *** |
| Cognitive Flexibility <-> Transportability | 0.20 | [0.13, 0.29] | <.001 | *** |
| Cognitive Flexibility -> Vividness | 0.18 | [0.094, 0.26] | <.001 | *** |
| Transportability -> Vividness | 0.34 | [0.26, 0.42] | <.001 | *** |
| Vividness -> Controllability | 0.60 | [0.53, 0.67] | <.001 | *** |
| Middle School OthFic | | | | |
| Reading -> Cognitive Flexibility | 0.026 | [−0.093, 0.15] | .66 |  |
| Reading -> Transportability | 0.056 | [−0.063, 0.18] | .35 |  |
| Cognitive Flexibility <-> Transportability | 0.22 | [0.13, 0.30] | <.001 | *** |
| Cognitive Flexibility -> Vividness | 0.18 | [0.094, 0.26] | <.001 | *** |
| Transportability -> Vividness | 0.34 | [0.26, 0.42] | <.001 | *** |
| Vividness -> Controllability | 0.60 | [0.53, 0.67] | <.001 | *** |
| Middle School OthNonFic | | | | |
| Reading -> Cognitive Flexibility | −0.089 | [−0.21, 0.035] | .16 |  |
| Reading -> Transportability | −0.059 | [−0.18, 0.065] | .35 |  |
| Cognitive Flexibility <-> Transportability | 0.22 | [0.14, 0.30] | <.001 | *** |
| Cognitive Flexibility -> Vividness | 0.18 | [0.094, 0.26] | <.001 | *** |
| Transportability -> Vividness | 0.34 | [0.26, 0.42] | <.001 | *** |
| Vividness -> Controllability | 0.60 | [0.53, 0.67] | <.001 | *** |
| Current Academic | | | | |
| Reading -> Cognitive Flexibility | 0.16 | [0.07, 0.25] | <.001 | *** |
| Reading -> Transportability | 0.09 | [−0.01, 0.18] | .06 |  |
| Cognitive Flexibility <-> Transportability | 0.20 | [0.12, 0.29] | <.001 | *** |
| Cognitive Flexibility -> Vividness | 0.18 | [0.09, 0.26] | <.001 | *** |
| Transportability -> Vividness | 0.34 | [0.26, 0.42] | <.001 | *** |
| Vividness -> Controllability | 0.60 | [0.53, 0.67] | <.001 | *** |
| Current Biography | | | | |
| Reading -> Cognitive Flexibility | 0.03 | [−0.08, 0.14] | .63 |  |
| Reading -> Transportability | −0.21 | [−0.32, −0.10] | <.001 | *** |
| Cognitive Flexibility <-> Transportability | 0.22 | [0.14, 0.31] | <.001 | *** |
| Cognitive Flexibility -> Vividness | 0.18 | [0.09, 0.26] | <.001 | *** |
| Transportability -> Vividness | 0.34 | [0.26, 0.42] | <.001 | *** |
| Vividness -> Controllability | 0.60 | [0.53, 0.67] | <.001 | *** |
| Current Comedy | | | | |
| Reading -> Cognitive Flexibility | −0.14 | [−0.25, −0.03] | .01 | * |
| Reading -> Transportability | −0.09 | [−0.20, 0.02] | .12 |  |
| Cognitive Flexibility <-> Transportability | 0.21 | [0.13, 0.30] | <.001 | *** |
| Cognitive Flexibility -> Vividness | 0.18 | [0.09, 0.26] | <.001 | *** |
| Transportability -> Vividness | 0.34 | [0.26, 0.42] | <.001 | *** |
| Vividness -> Controllability | 0.60 | [0.53, 0.67] | <.001 | *** |
| Current Cookbooks | | | | |
| Reading -> Cognitive Flexibility | −0.08 | [−0.18, 0.03] | .14 |  |
| Reading -> Transportability | −0.10 | [−0.20, 0.00] | .05 |  |
| Cognitive Flexibility <-> Transportability | 0.21 | [0.13, 0.30] | <.001 | *** |
| Cognitive Flexibility -> Vividness | 0.18 | [0.09, 0.26] | <.001 | *** |
| Transportability -> Vividness | 0.34 | [0.26, 0.42] | <.001 | *** |
| Vividness -> Controllability | 0.60 | [0.53, 0.67] | <.001 | *** |
| Current Crime | | | | |
| Reading -> Cognitive Flexibility | −0.04 | [−0.15, 0.07] | .51 |  |
| Reading -> Transportability | 0.10 | [−0.01, 0.21] | .08 |  |
| Cognitive Flexibility <-> Transportability | 0.22 | [0.14, 0.31] | <.001 | *** |
| Cognitive Flexibility -> Vividness | 0.18 | [0.09, 0.26] | <.001 | *** |
| Transportability -> Vividness | 0.34 | [0.26, 0.42] | <.001 | *** |
| Vividness -> Controllability | 0.60 | [0.53, 0.67] | <.001 | *** |
| Current Essays | | | | |
| Reading -> Cognitive Flexibility | 0.13 | [0.04, 0.23] | <.001 | ** |
| Reading -> Transportability | 0.04 | [−0.05, 0.13] | .40 |  |
| Cognitive Flexibility <-> Transportability | 0.21 | [0.13, 0.30] | <.001 | *** |
| Cognitive Flexibility -> Vividness | 0.18 | [0.09, 0.26] | <.001 | *** |
| Transportability -> Vividness | 0.34 | [0.26, 0.42] | <.001 | *** |
| Vividness -> Controllability | 0.60 | [0.53, 0.67] | <.001 | *** |
| Current Fantasy | | | | |
| Reading -> Cognitive Flexibility | 0.09 | [−0.01, 0.19] | .09 |  |
| Reading -> Transportability | 0.22 | [0.13, 0.32] | <.001 | *** |
| Cognitive Flexibility <-> Transportability | 0.20 | [0.12, 0.29] | <.001 | *** |
| Cognitive Flexibility -> Vividness | 0.18 | [0.09, 0.26] | <.001 | *** |
| Transportability -> Vividness | 0.34 | [0.26, 0.42] | <.001 | *** |
| Vividness -> Controllability | 0.60 | [0.53, 0.67] | <.001 | *** |
| Current Graphic | | | | |
| Reading -> Cognitive Flexibility | −0.20 | [−0.30, −0.10] | <.001 | *** |
| Reading -> Transportability | −0.03 | [−0.13, 0.07] | .55 |  |
| Cognitive Flexibility <-> Transportability | 0.21 | [0.13, 0.30] | <.001 | *** |
| Cognitive Flexibility -> Vividness | 0.18 | [0.09, 0.26] | <.001 | *** |
| Transportability -> Vividness | 0.34 | [0.26, 0.42] | <.001 | *** |
| Vividness -> Controllability | 0.60 | [0.53, 0.67] | <.001 | *** |
| Current Health | | | | |
| Reading -> Cognitive Flexibility | 0.00 | [−0.11, 0.10] | .98 |  |
| Reading -> Transportability | −0.19 | [−0.29, −0.09] | <.001 | *** |
| Cognitive Flexibility <-> Transportability | 0.22 | [0.14, 0.30] | <.001 | *** |
| Cognitive Flexibility -> Vividness | 0.18 | [0.09, 0.26] | <.001 | *** |
| Transportability -> Vividness | 0.34 | [0.26, 0.42] | <.001 | *** |
| Vividness -> Controllability | 0.60 | [0.53, 0.67] |  |  |
| Current HistFic | | | | |
| Reading -> Cognitive Flexibility | −0.05 | [−0.17, 0.07] | .42 |  |
| Reading -> Transportability | −0.02 | [−0.14, 0.10] | .78 |  |
| Cognitive Flexibility <-> Transportability | 0.22 | [0.13, 0.31] | <.001 | *** |
| Cognitive Flexibility -> Vividness | 0.18 | [0.09, 0.26] | <.001 | *** |
| Transportability -> Vividness | 0.34 | [0.26, 0.42] | <.001 | *** |
| Vividness -> Controllability | 0.60 | [0.53, 0.67] | <.001 | *** |
| Current Hist | | | | |
| Reading -> Cognitive Flexibility | 0.00 | [−0.11, 0.11] | .99 |  |
| Reading -> Transportability | −0.13 | [−0.24, −0.03] | .01 | * |
| Cognitive Flexibility <-> Transportability | 0.22 | [0.14, 0.30] | <.001 | *** |
| Cognitive Flexibility -> Vividness | 0.18 | [0.09, 0.26] | <.001 | *** |
| Transportability -> Vividness | 0.34 | [0.26, 0.42] | <.001 | *** |
| Vividness -> Controllability | 0.60 | [0.53, 0.67] | <.001 | *** |
| Current Horror | | | | |
| Reading -> Cognitive Flexibility | −0.09 | [−0.20, 0.02] | .10 |  |
| Reading -> Transportability | −0.10 | [−0.21, 0.00] | .06 |  |
| Cognitive Flexibility <-> Transportability | 0.21 | [0.13, 0.30] | <.001 | *** |
| Cognitive Flexibility -> Vividness | 0.18 | [0.09, 0.26] | <.001 | *** |
| Transportability -> Vividness | 0.34 | [0.26, 0.42] | <.001 | *** |
| Vividness -> Controllability | 0.60 | [0.53, 0.67] | <.001 | *** |
| Current LitFic | | | | |
| Reading -> Cognitive Flexibility | 0.10 | [−0.01, 0.21] | .08 |  |
| Reading -> Transportability | 0.12 | [0.01, 0.23] | .03 | * |
| Cognitive Flexibility <-> Transportability | 0.21 | [0.13, 0.30] | <.001 | *** |
| Cognitive Flexibility -> Vividness | 0.18 | [0.09, 0.26] | <.001 | *** |
| Transportability -> Vividness | 0.34 | [0.26, 0.42] | <.001 | *** |
| Vividness -> Controllability | 0.60 | [0.53, 0.67] | <.001 | *** |
| Current Memoir | | | | |
| Reading -> Cognitive Flexibility | 0.05 | [−0.06, 0.17] | .34 |  |
| Reading -> Transportability | −0.09 | [−0.20, 0.02] | .12 |  |
| Cognitive Flexibility <-> Transportability | 0.22 | [0.14, 0.31] | <.001 | *** |
| Cognitive Flexibility -> Vividness | 0.18 | [0.09, 0.26] | <.001 | *** |
| Transportability -> Vividness | 0.34 | [0.26, 0.42] | <.001 | *** |
| Vividness -> Controllability | 0.60 | [0.53, 0.67] | <.001 | *** |
| Current Poetry | | | | |
| Reading -> Cognitive Flexibility | 0.02 | [−0.08, 0.13] | .69 |  |
| Reading -> Transportability | 0.02 | [−0.08, 0.12] | .70 |  |
| Cognitive Flexibility <-> Transportability | 0.22 | [0.13, 0.31] | <.001 | *** |
| Cognitive Flexibility -> Vividness | 0.18 | [0.09, 0.26] | <.001 | *** |
| Transportability -> Vividness | 0.34 | [0.26, 0.42] | <.001 | *** |
| Vividness -> Controllability | 0.60 | [0.53, 0.67] | <.001 | *** |
| Current Romance | | | | |
| Reading -> Cognitive Flexibility | 0.01 | [−0.10, 0.12] | .85 |  |
| Reading -> Transportability | 0.31 | [0.20, 0.41] | <.001 | *** |
| Cognitive Flexibility <-> Transportability | 0.22 | [0.14, 0.30] | <.001 | *** |
| Cognitive Flexibility -> Vividness | 0.18 | [0.09, 0.26] | <.001 | *** |
| Transportability -> Vividness | 0.34 | [0.26, 0.42] | <.001 | *** |
| Vividness -> Controllability | 0.60 | [0.53, 0.67] | <.001 | *** |
| Current SciFi | | | | |
| Reading -> Cognitive Flexibility | 0.10 | [−0.01, 0.22] | .07 |  |
| Reading -> Transportability | 0.13 | [0.02, 0.24] | .02 | * |
| Cognitive Flexibility <-> Transportability | 0.21 | [0.13, 0.30] | <.001 | *** |
| Cognitive Flexibility -> Vividness | 0.18 | [0.09, 0.26] | <.001 | *** |
| Transportability -> Vividness | 0.34 | [0.26, 0.42] | <.001 | *** |
| Vividness -> Controllability | 0.60 | [0.53, 0.67] | <.001 | *** |
| Current SelfHelp | | | | |
| Reading -> Cognitive Flexibility | 0.02 | [−0.08, 0.13] | .64 |  |
| Reading -> Transportability | −0.05 | [−0.15, 0.06] | .37 |  |
| Cognitive Flexibility <-> Transportability | 0.22 | [0.14, 0.31] | <.001 | *** |
| Cognitive Flexibility -> Vividness | 0.18 | [0.09, 0.26] | <.001 | *** |
| Transportability -> Vividness | 0.34 | [0.26, 0.42] | <.001 | *** |
| Vividness -> Controllability | 0.60 | [0.53, 0.67] | <.001 | *** |
| Current Spirituality | | | | |
| Reading -> Cognitive Flexibility | −0.03 | [−0.13, 0.07] | .51 |  |
| Reading -> Transportability | −0.09 | [−0.19, 0.01] | .07 |  |
| Cognitive Flexibility <-> Transportability | 0.21 | [0.13, 0.30] | <.001 | *** |
| Cognitive Flexibility -> Vividness | 0.18 | [0.09, 0.26] | <.001 | *** |
| Transportability -> Vividness | 0.34 | [0.26, 0.42] | <.001 | *** |
| Vividness -> Controllability | 0.60 | [0.53, 0.67] | <.001 | *** |
| Current Travel | | | | |
| Reading -> Cognitive Flexibility | −0.17 | [−0.29, −0.06] | <.001 | ** |
| Reading -> Transportability | −0.15 | [−0.26, −0.03] | .01 | * |
| Cognitive Flexibility <-> Transportability | 0.20 | [0.12, 0.29] | <.001 | *** |
| Cognitive Flexibility -> Vividness | 0.18 | [0.09, 0.26] | <.001 | *** |
| Transportability -> Vividness | 0.34 | [0.26, 0.42] | <.001 | *** |
| Vividness -> Controllability | 0.60 | [0.53, 0.67] | <.001 | *** |
| Current Urban | | | | |
| Reading -> Cognitive Flexibility | −0.26 | [−0.37, −0.15] | <.001 | *** |
| Reading -> Transportability | −0.18 | [−0.29, −0.07] | <.001 | ** |
| Cognitive Flexibility <-> Transportability | 0.19 | [0.11, 0.27] | <.001 | *** |
| Cognitive Flexibility -> Vividness | 0.18 | [0.09, 0.26] | <.001 | *** |
| Transportability -> Vividness | 0.34 | [0.26, 0.42] | <.001 | *** |
| Vividness -> Controllability | 0.60 | [0.53, 0.67] | <.001 | *** |
| Current YA | | | | |
| Reading -> Cognitive Flexibility | −0.10 | [−0.20, 0.01] | .07 |  |
| Reading -> Transportability | 0.14 | [0.03, 0.24] | .01 | ** |
| Cognitive Flexibility <-> Transportability | 0.23 | [0.14, 0.31] | <.001 | *** |
| Cognitive Flexibility -> Vividness | 0.18 | [0.09, 0.26] | <.001 | *** |
| Transportability -> Vividness | 0.34 | [0.26, 0.42] | <.001 | *** |
| Vividness -> Controllability | 0.60 | [0.53, 0.67] | <.001 | *** |
| Current OthFic | | | | |
| Reading -> Cognitive Flexibility | 0.09 | [−0.02, 0.21] | .09 |  |
| Reading -> Transportability | 0.02 | [−0.09, 0.13] | .69 |  |
| Cognitive Flexibility <-> Transportability | 0.22 | [0.13, 0.30] | <.001 | *** |
| Cognitive Flexibility -> Vividness | 0.18 | [0.09, 0.26] | <.001 | *** |
| Transportability -> Vividness | 0.34 | [0.26, 0.42] | <.001 | *** |
| Vividness -> Controllability | 0.60 | [0.53, 0.67] | <.001 | *** |
| Current OthNonFic | | | | |
| Reading -> Cognitive Flexibility | 0.03 | [−0.08, 0.14] | .55 |  |
| Reading -> Transportability | −0.07 | [−0.18, 0.04] | .22 |  |
| Cognitive Flexibility <-> Transportability | 0.22 | [0.14, 0.31] | <.001 | *** |
| Cognitive Flexibility -> Vividness | 0.18 | [0.09, 0.26] | <.001 | *** |
| Transportability -> Vividness | 0.34 | [0.26, 0.42] | <.001 | *** |
| Vividness -> Controllability | 0.60 | [0.53, 0.67] | <.001 | *** |

Table S3

*Fit Indices for Alternate Path Models, Middle-School Fiction*

|  | AIC | BIC | CFI | TLI | RMSEA [95% CI] |
| --- | --- | --- | --- | --- | --- |
| In-Text Model | 11,338.80 | 11378.34 | 0.987 | 0.958 | 0.069 [0.043, 0.095] |
| Alternate Model 1 | 11,662.90 | 11,706.16 | 0.862 | -0.034 | 0.34 [0.31, 0.38] |
| Alternate Model 2 | 11,430.71 | 11,466.38 | 0.946 | 0.885 | 0.12 [0.095, 0.14] |
| Alternate Model 3 | 11,443.65 | 11,479.32 | 0.941 | 0.873 | 0.12 [0.10, 0.14] |
| Alternate Model 4 | 11,364.61 | 11,404.15 | 0.976 | 0.925 | 0.092 [0.068, 0.12] |

Note: AIC = Akaike Information Criterion, BIC = Bayesian Information Criterion, CFI = Comparative Fit Index, TLI = Tucker-Lewis Index, RMSEA = Root Mean Square Error of Approximation. For path diagrams of alternate models, see Figure S1.


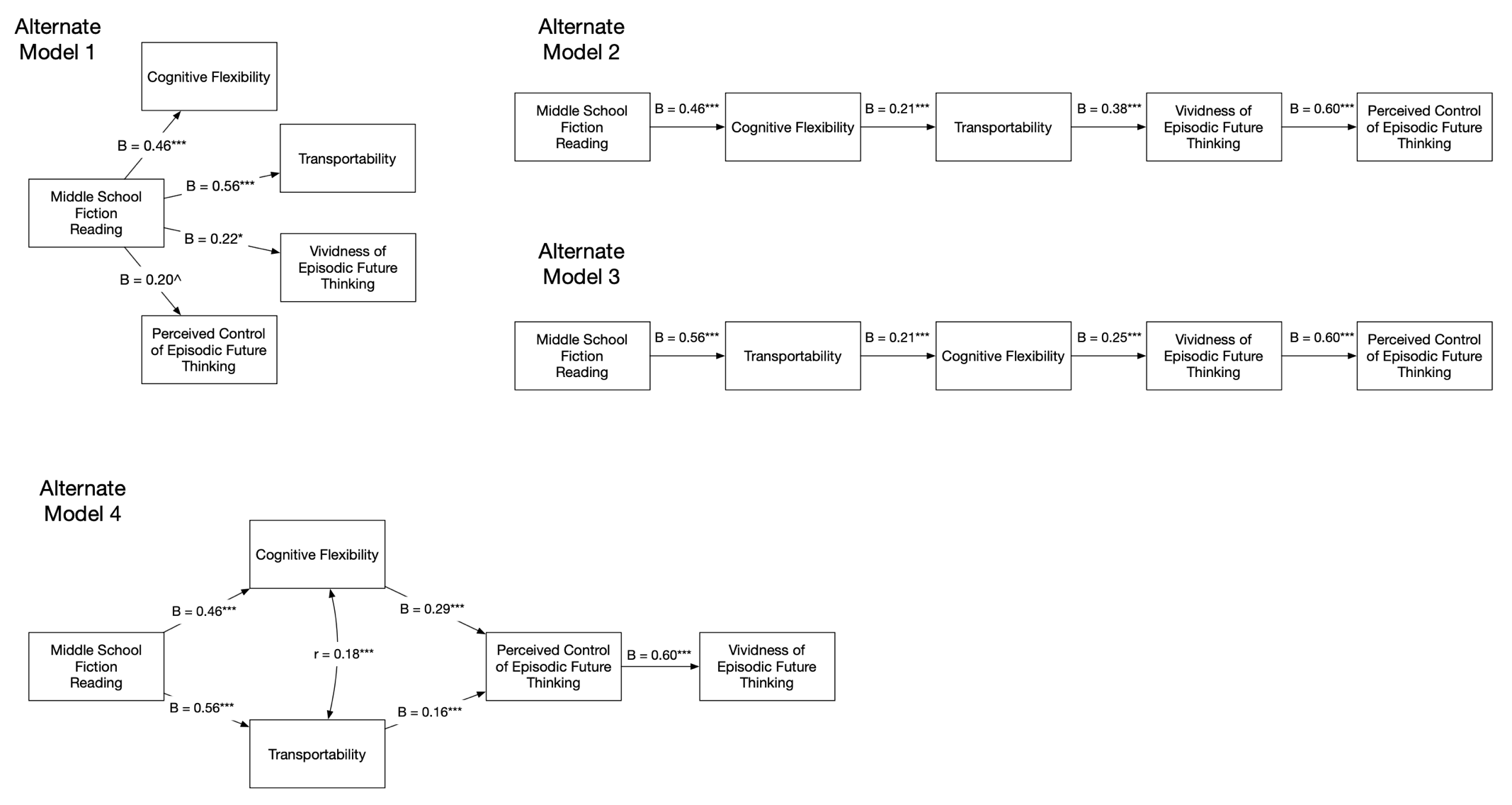


*Figure S1.* Path diagrams for alternate models.

All models contain the same controls as in the main-text (total middle school reading, total present day reading, present-day fiction reading, parental education, and gender). ^*p* < .06, **p* < .05, ****p* < .001.
